# Impact of Inadequate Calorie Intake on Mortality and Hospitalization in Stable Patients with Chronic Heart Failure

**DOI:** 10.3390/nu13030874

**Published:** 2021-03-08

**Authors:** Yoshikuni Obata, Naoya Kakutani, Shintaro Kinugawa, Arata Fukushima, Takashi Yokota, Shingo Takada, Taisuke Ono, Takeshi Sota, Yoshiharu Kinugasa, Masashige Takahashi, Hisashi Matsuo, Ryuichi Matsukawa, Ichiro Yoshida, Isao Yokota, Kazuhiro Yamamoto, Miyuki Tsuchihashi-Makaya

**Affiliations:** 1 Department of Cardiovascular Medicine, Faculty of Medicine, Graduate School of Medicine, Hokkaido University, Sapporo 060-8638, Japan; obata4492@yahoo.co.jp (Y.O.); kakutaninaoya@gmail.com (N.K.); arata.fukushima@gmail.com (A.F.); t-yokota@med.hokudai.ac.jp (T.Y.); s-takada@hokusho-u.ac.jp (S.T.); 2 Department of Cardiovascular Medicine, Faculty of Medical Sciences, Kyusyu University, Fukuoka 812-8582, Japan; 3 Clinical Research and Medical Innovation Center, Hokkaido University Hospital, Sapporo 060-8648, Japan; 4 Department of Cardiology, Kitami Red Cross Hospital, Kitami 090-8666, Japan; ono_taisuke@kitami.jrc.or.jp; 5 Division of Rehabilitation, Tottori University Hospital, Tottori 683-8504, Japan; tsota@med.tottori-u.ac.jp; 6 Department of Cardiovascular Medicine and Endocrinology and Metabolism, Faculty of Medicine, Tottori University, Tottori 683-8503, Japan; ykinugasa-circ@umin.ac.jp (Y.K.); ykazuhiro@med.tottori-u.ac.jp (K.Y.); 7 Department of Cardiology, Kushiro City General Hospital, Kushiro 085-0822, Japan; circ.masashiget@gmail.com; 8 Department of Cardiology, Keiwakai Ebetsu Hospital, Ebetsu 069-0817, Japan; matsuo@keiwakai-ebetsu.or.jp; 9 Division of Cardiology, Cardiovascular and Aortic Center, Saiseikai Fukuoka General Hospital, Fukuoka 810-0001, Japan; matukawa@cardiol.med.kyushu-u.ac.jp; 10 Department of Cardiology, Obihiro Kyokai Hospital, Obihiro 080-0805, Japan; i-yoshida@obihiro-kyokai-hsp.jp; 11 Department of Biostatistics, Faculty of Medicine, Graduate School of Medicine, Hokkaido University, Sapporo 060-8638, Japan; yokotai@pop.med.hokudai.ac.jp; 12 School of Nursing, Kitasato University, Sagamihara 252-0373, Japan; miyuki-m@nrs.kitasato-u.ac.jp

**Keywords:** calorie intake, heart failure, hospitalization, malnutrition, mortality

## Abstract

Malnutrition is highly prevalent in patients with heart failure (HF), but the precise impact of dietary energy deficiency on HF patients’ clinical outcomes is not known. We investigated the associations between inadequate calorie intake and adverse clinical events in 145 stable outpatients with chronic HF who had a history of hospitalization due to worsening HF. To assess the patients’ dietary pattern, we used a brief self-administered diet-history questionnaire (BDHQ). Inadequate calorie intake was defined as <60% of the estimated energy requirement. In the total chronic HF cohort, the median calorie intake was 1628 kcal/day. Forty-four patients (30%) were identified as having an inadequate calorie intake. A Kaplan–Meier analysis revealed that the patients with inadequate calorie intake had significantly worse clinical outcomes including all-cause death and HF-related hospitalization during the 1-year follow-up period versus those with adequate calorie intake (20% vs. 5%, *p* < 0.01). A multivariate logistic regression analysis showed that inadequate calorie intake was an independent predictor of adverse clinical events after adjustment for various factors that may influence patients’ calorie intake. Among patients with chronic HF, inadequate calorie intake was associated with an increased risk of all-cause mortality and rehospitalization due to worsening HF. However, our results are preliminary and larger studies with direct measurements of dietary calorie intake and total energy expenditure are needed to clarify the intrinsic nature of this relationship.

## 1. Introduction

Heart failure (HF) is common in adults and is associated with increased morbidity and mortality. Its prevalence is increasing due to the aging of the population in many countries [1]. Despite recent advances in pharmacological and non-pharmacological treatments for HF, the prognosis of individuals with chronic HF remains poor, and diet and exercise interventions are thus recognized as essential treatments for the prevention of HF progression.

Although obesity is a risk of incident HF, a low body mass index (BMI) is more closely associated with poor clinical outcomes in chronic HF patients, in a phenomenon known as the obesity paradox [2,3]. As one of the possible mechanisms of this paradox, malnutrition is a recent focus of attention among healthcare providers who are engaged in HF management. Malnutrition is highly prevalent in patients with chronic HF, and it increases their risk of death and hospitalization [4]. Patients with chronic HF have been demonstrated to have an increased energy expenditure compared to healthy sedentary subjects, but HF patients’ dietary energy intake is often insufficient to meet their energy requirements for daily activities, even in a stable condition [5]. The negative energy balance leads to a catabolic state and causes protein–energy malnutrition, which results in muscle wasting and sarcopenia [6,7]. In addition, dietary guidance for HF patients has traditionally focused on reducing their salt and fluid intake; the patients’ intake of dietary nutrients has tended to be less of a concern [8]. Restrictive diets for HF patients may cause a reduced intake of macronutrients and micronutrients, leading to increased morbidity and mortality [8].

We conducted the present study to determine whether calorie intake that is inadequate for the energy needed for daily activities is associated with adverse clinical events including all-cause death and HF-related hospitalization in stable patients with chronic HF. The patients’ daily calorie intake was calculated by a brief self-administered diet-history questionnaire (BDHQ), which is a well-validated questionnaire for determining a patient’s dietary pattern.

## 2. Materials and Methods

### 2.1. Study Design

This study was part of a multicenter, prospective observational investigation of the effects of dietary patterns on clinical outcomes in patients with chronic HF, and thus some of the data used herein were obtained from the same patients whose data were published previously but in a different context [9]. The study was approved by the ethics committees of Hokkaido University Hospital (approval no. 012-0224) and the other nine participating research institutes—Hakodate National Hospital, Hikone Municipal Hospital, Kitami Red Cross Hospital, Keiwakai Ebetsu Hospital, Kushiro City General Hospital, Obihiro Kyokai Hospital, Otaru Kyokai Hospital, Saiseikai Fukuoka General Hospital, and Tottori University Hospital. The study was conducted in accordance with the ethical principles described in the Declaration of Helsinki. Written informed consent was obtained from each patient before his or her participation in the study.

### 2.2. Patients

A total of 145 stable patients with chronic HF who were regularly visiting an outpatient ward for >1 month were enrolled between December 2012 and September 2014. These patients had a history of hospitalization due to worsening HF at least once within the 5 years before enrollment. The exclusion criteria included nephrotic syndrome, liver cirrhosis, cancer, a history of gastrointestinal surgery within the prior 3 months, or poorly controlled diabetes, i.e., glycosylated hemoglobin (HbA1c) >7.0%. We also excluded patients who were taking steroids or antidepressants, which could influence their appetite.

### 2.3. Study Protocol

At baseline, the patients underwent clinical and anthropometric measurements, blood testing, echocardiography, a 6-min walk test to assess exercise capacity, and the evaluation of their dietary pattern and calorie intake. The patients were then followed up for 1 year to evaluate adverse clinical events including all-cause death and hospitalization due to worsening HF.

### 2.4. Anthropometric Measurements

To assess the patients’ muscle mass, we measured the circumferences of the upper arm and the thigh at the level of the muscle belly.

### 2.5. Laboratory Measurements

After blood collection, the patients’ hemoglobin, serum albumin, HbA1c, and plasma levels of B-type natriuretic peptide (BNP) were determined by routine in-house analyses. The estimated glomerular filtration rate (eGFR) was calculated from the serum creatinine values and the patient’s age with the use of the Japanese equation [10]: eGFR = 194 × (serum creatinine, mg/dL)^−1.094^ × (age, years)^−0.287^ × (0.739 if female).

### 2.6. Assessment of Dietary Calorie Intake

Each patient’s dietary pattern was evaluated using a BDHQ adjusted to typical Japanese diets. The BDHQ is a four-page fixed-portion questionnaire that calculates the frequency of the consumption of selected foods to estimate the intake of 58 food and beverage items during the preceding month, as described [11,12]. The BDHQ consists of five sections—(1) the intake frequency of food and nonalcoholic beverage items, (2) the daily intake of rice and miso soup, (3) the frequency of alcoholic beverage consumption and the amount per drink, (4) usual cooking methods, and (5) general dietary behavior. The dietary calorie intake was calculated as the sum of each energy conversion factor from the fats, proteins, and carbohydrates whose amount is estimated using the BDHQ, as described previously [13,14]. Dietary salt intake was estimated according to the diet history method using the quantitative information. In this estimation, intakes of table salt and salt-containing seasoning at the table, calculated using the qualitative information of general dietary behavior, were also considered, as described [12].

### 2.7. Estimation of the Dietary Calorie Requirement

The dietary calorie requirement was estimated using the Japanese Dietary Reference Intakes published by the Ministry of Health, Labour and Welfare (Japan) in 2015, as described previously [15,16]. Briefly, each patient’s estimated dietary calorie requirement was determined in consideration of his or her age, gender, and physical activity level (low, moderate, or high). Since most of the patients were in a stable condition with New York Heart Association (NYHA) functional class I or II (normal or mild HF) and all the patients were ambulant and regularly visited an outpatient ward, the daily calorie requirement was estimated with the assumption that all of the patients were engaged in moderate physical activity (categorized as level II). This level requires the ability to do self-care activities (e.g., washing and dressing) and walk outside without any support. We then calculated the dietary energy adequacy (%) as the ratio of the individual patient’s daily calorie intake to the estimated daily calorie requirement.

### 2.8. Assessment of Nutritional Status

Each patient’s nutritional status was assessed by determining his or her controlling nutritional status (CONUT) score [17] and score on a geriatric nutritional risk index (GNRI) [18]. Briefly, the CONUT score was calculated based on the serum albumin level, total peripheral lymphocyte count, and total cholesterol level, and the scores are classified into normal (0–1 points), mild risk (2–4), moderate risk (5–8), and severe risk (9–12) of malnutrition. The GNRI was calculated from the patient’s BMI and albumin concentration according to the modified version—GNRI = 14.89 × serum albumin (g/dL) + 41.7 × BMI/22. The GNRI values are classified into four grades of malnutrition-related risk—major risk (GNRI < 82), moderate risk (GNRI 82–91), low risk (GNRI 92–98), and no risk (GNRI > 98).

### 2.9. Statistical Analyses

Continuous variables are expressed as medians (interquartile range), and categorical variables are expressed as numbers (percentages). We divided the 145 patients into two groups based on their dietary calorie intake adequacy—the adequate calorie intake group (dietary calorie intake adequacy ≥60%; *N* = 101) and the inadequate calorie intake group (dietary calorie intake adequacy <60%; *N* = 44). The cut-off value of dietary calorie intake adequacy rate (60%) was predetermined by the results of the multivariate analysis. Continuous variables were compared between these groups with a Mann–Whitney U-test, and the χ^2^-test was used for group comparisons of categorical variables. We performed a multivariate analysis to identify the decrease in dietary calorie intake adequacy that independently predicts adverse clinical events in chronic HF patients with other confounding factors that may influence dietary calorie intake, including age, BMI, NYHA functional class III, diabetes, left ventricular ejection fraction (LVEF), serum albumin, eGFR, and log BNP. The odds ratios (ORs) and 95% confidence intervals (CIs) were calculated for each variable from the logistic regression model. A Kaplan–Meier analysis with log-rank test was performed to assess the rates of all-cause death and rehospitalization due to worsening HF for 1 year. All analyses were performed using JMP Pro 13.1.0 software (SAS Institute, Cary, NC, USA). Probability (*p*)-values < 0.05 were considered significant.

## 3. Results

### 3.1. Characteristics of the Total Chronic HF Cohort

**Figure 1:**
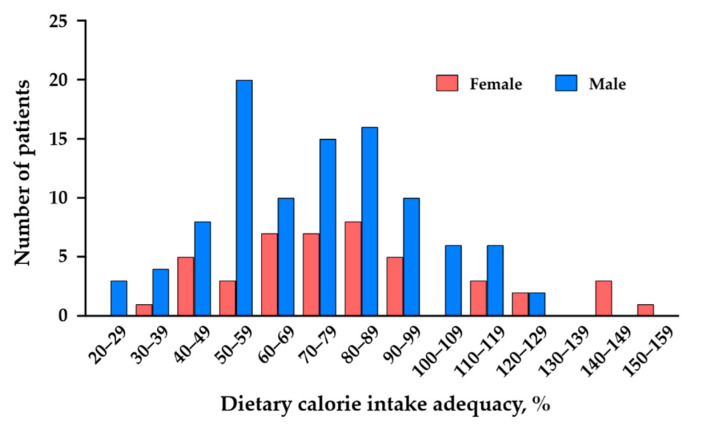
Distribution of dietary calorie intake adequacy rate of the female and male patients with chronic HF.

The characteristics of the total chronic HF cohort (*N* = 145) are summarized in Table 1. The median age of the patients with chronic HF was 67 years, and the median BMI was 22.9 kg/m^2^. We recruited stable outpatients with chronic HF, and 90% of the patients had an NYHA functional class I or II. The median LVEF was 45%, and both HF patients with a reduced LVEF and those with a preserved LVEF were included in this cohort. The majority of the chronic HF patients were being treated with an angiotensin-converting enzyme (ACE) inhibitor or an angiotensin II receptor blocker (ARB) and a β-blocker. For the total chronic HF cohort, the median value of dietary calorie intake was 1628 kcal/day and the dietary calorie intake adequacy rate was 75%. The distribution of the dietary calorie intake adequacy rates of the patients is shown in Figure 1.

### 3.2. Characteristics of the Chronic HF Patients with and without Adequate Calorie Intake

We divided the total chronic HF cohort into two groups—the adequate calorie intake group (*N* = 101) and the inadequate calorie intake group (*N* = 44). Inadequate calorie intake was defined as <60% of the estimated calorie requirement according to the results of the multivariate analysis. The baseline data of each group are summarized in Table 2. The median age of the chronic HF patients with inadequate calorie intake was younger than that of the patients with adequate calorie intake, but there was no significant difference in BMI or muscle mass (i.e., upper arm and thigh circumferences) between the groups. The percentage of diabetes was greater in the inadequate calorie intake group compared to the adequate calorie intake group. The LVEF, a parameter of LV systolic function, was significantly lower in the chronic HF patients with inadequate calorie intake. Renal function (i.e., eGFR) was more often impaired in the patients with inadequate calorie intake.

The nutritional parameters including the CONUT score and GNRI value were similar between the two groups. As expected, the chronic HF patients with inadequate calorie intake had a reduced daily calorie intake compared to those with an adequate calorie intake. In addition, most of foods and nutrients were less frequently consumed by the patient with inadequate calorie intake (Supplementary Tables S1 and S2). The daily salt intake was significantly reduced in the inadequate calorie intake group compared to the adequate calorie intake group (median (1st–3rd quartile range) 6.6 (5.4–8.3) vs. 10.2 (8.8–13.3) g/day, *p*<0.01) (Supplementary Table S2).

### 3.3. Adverse Clinical Events

**Figure 2:**
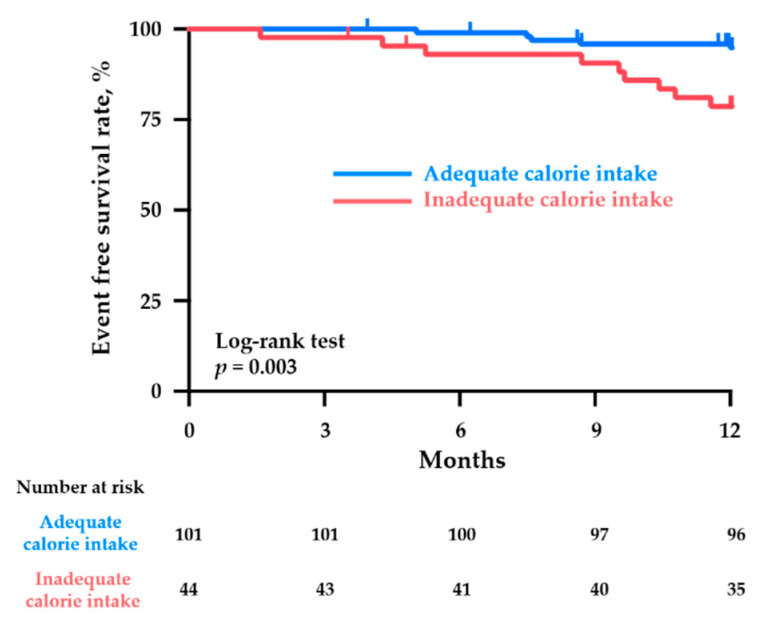
Kaplan–Meier curves for the cumulative event (all-cause death and HF-related hospitalization)-free ratio in the chronic HF patients with an adequate calorie intake (dietary calorie intake adequacy ≥60%) and those with an inadequate calorie intake (dietary calorie intake adequacy <60%).

During the 1-year follow-up period, the combined clinical events of all-cause death and HF-related hospitalization occurred in 14 patients (10%) (four deaths and 10 hospitalizations). The Kaplan–Meier analysis revealed that the patients with an inadequate calorie intake had a significantly higher risk of adverse clinical events than those with an adequate calorie intake (20% vs. 5%, respectively; *p <* 0.01) (Figure 2).

### 3.4. Predictors of Adverse Clinical Events in Patients with Chronic HF

The results of the multivariate analysis revealed that after the adjustment for age, BMI, NYHA functional class III, LVEF, serum albumin, eGFR, and log BNP, inadequate calorie intake defined as <60% of the estimated calorie requirement was a significantly independent predictor of adverse clinical events including all-cause death and HF-related hospitalization over 1 year in the patients with chronic HF (Table 3).

## 4. Discussion

In the present cohort of 145 patients with chronic HF, the inadequate calorie intake group had a significantly higher risk of adverse clinical events including all-cause death and hospitalization due to worsening HF for the 1-year follow-up period compared to the adequate calorie intake group, when we defined inadequate calorie intake as <60% of estimated calorie requirement. The multivariate logistic regression analysis showed that dietary calorie intake adequacy <60% was an independent predictor of worse clinical outcomes after adjustment for age, BMI, NYHA functional class III, diabetes, LVEF, serum albumin, eGFR, and log BNP in chronic HF patients. To the best of our knowledge, this is the first study that revealed the impact of dietary energy deficiency on mortality and hospitalization in stable outpatients with chronic HF.

Although several nutritional assessment tools such as the CONUT score and the GNRI are used in clinical practice, we here focused on a questionnaire-based assessment of the daily calorie intake in patients with chronic HF. All of the patients were in stable condition at baseline, and most of them were categorized as having no risk or only a mild risk of malnutrition when they were evaluated using the CONUT score or the GNRI. The malnutrition risk scores calculated by these nutritional assessment tools did not differ between the patients with inadequate calorie intake and those with adequate calorie intake. Accordingly, our present finding that the lowered dietary calorie intake adequacy was associated with increased risks of death and hospitalization in chronic HF patients indicates that the dietary calorie intake can be a useful nutritional assessment tool to detect the early stage of malnutrition in stable patients with chronic HF.

Cardiac cachexia, characterized by weight loss, is a major contributor to a poor prognosis in chronic HF patients [19]. The negative energy balance caused by an inadequate dietary calorie intake that does not support energy needs may lead to protein breakdown, which results in muscle wasting and sarcopenia [6,7]. Although in the present investigation, the BMI and muscle mass measured at baseline were not reduced in the patients with inadequate calorie intake, a sustained dietary energy deficiency may contribute to the future onset of cardiac cachexia and sarcopenia.

Our analyses revealed that the daily intakes of macronutrients and micronutrients were significantly decreased in the chronic HF patients with inadequate calorie intake, and this pattern might lead to worse clinical outcomes. Deficiencies of micronutrients such as minerals and vitamins have been reported to potentially impair cardiac and systemic functional capacity, which results in reduced quality of life and poor prognosis [20,21]. Oxidative stress also plays a crucial role in the progression of HF [22,23,24,25], and in the present cohort, the patients with inadequate calorie intakes had lowered consumptions of antioxidative nutrients such as vitamin C, vitamin E, and carotenoids, which might also affect the increased rate of adverse clinical events.

Although we could not quantify the patient’s appetite, intestinal congestion may cause appetite loss, which results in inadequate calorie intake in chronic HF patients. It is reported that cachectic patients with chronic HF had a larger bowel wall thickness (i.e., intestinal congestion) in the entire colon [26]. In addition, decreased hunger sensation and HF-related symptoms (such as fatigue, nausea, and anxiety) may be related to reduced calorie intake in chronic HF patients [27]. Taken together, digestive disturbance and HF-related symptoms may affect inadequate calorie intake in these patients.

Dietary salt restriction is widely recommended to HF patients as a dietary intervention. Unexpectedly, we observed that the daily salt intake was significantly lower in the patients with an inadequate calorie intake, who had a higher risk of adverse clinical events. It has been reported that strict adherence to salt restriction may lead to appetite loss and reduced calorie intake, which results in a dietary nutritional deficiency in chronic HF patients [28]. Accordingly, more comprehensive dietary interventions in consideration of dietary calorie intake adequacy and nutritional balance as well as salt restriction are necessary for the prevention of HF progression.

There are some study limitations to consider. First, the number of patients with inadequate calorie intake was small (*N* = 44). Second, the dietary calorie and nutritional intake were evaluated on the basis of the patient’s self-reported information about dietary patterns, and we thus could not directly measure their dietary calorie intake. In addition, each patient’s calorie requirement was estimated using the Japanese Dietary Reference Intakes for the general population. Because HF patients’ energy expenditure is likely to be higher than that of healthy subjects, we cannot completely exclude the possibility that our patients’ calorie requirement might be underestimated. Direct measurements of calorie intake and energy expenditure considering the patients’ daily physical activity level might increase the accuracy of the estimations of dietary calorie intake adequacy. Finally, we did not evaluate the social, economic, or environmental conditions of patients, although these factors may also affect dietary calorie intake.

## 5. Conclusions

In stable patients with chronic HF, inadequate dietary calorie intake was independently associated with an increased risk of adverse clinical events including all-cause death and hospitalization due to worsening HF.

## Figures and Tables

**Table 1:** Characteristics of total chronic heart failure (HF) cohort (*N* = 145).

Demographic Findings:	
Age, year	67 (60–77)
Female	45 (31%)
BMI, kg/m^2^	22.9 (20.5–25.7)
Upper arm circumference, cm	27.5 (24.9–29.7)
Thigh circumference, cm	44.4 (40.8–47.0)
NYHA functional class:	
I–II	130 (90%)
III	15 (10%)
Primary cause of HF:	
Ischemic cause	46 (32%)
Dilated cardiomyopathy	45 (31%)
Others	54 (37%)
Hypertension	80 (55%)
Diabetes	38 (26%)
Dyslipidemia	100 (69%)
Echocardiographic findings:	
LVEF, %	45 (30–56)
Laboratory measurements:	
Hemoglobin, g/dL	13.3 (11.9–14.3)
Serum albumin, g/dL	4.2 (3.9–4.4)
eGFR, mL/min/1.73 m^2^	54.2 (40.3–67.7)
HbA1c, %	5.8 (5.6–6.2)
Plasma BNP, pg/mL	154 (76–368)
Medications:	
ACE inhibitor or ARB	110 (76%)
β-blocker	128 (88%)
MRA	83 (57%)
Statin	69 (48%)
6-min walk test, m	433 (349–499)
Nutritional assessments:	
CONUT score	2 (1–2)
GNRI	106 (100–113)
Dietary calorie intake, kcal/day	1628 (1274–1996)
Estimated calorie requirement, kcal/day	2300 (1956–2425)
Dietary calorie intake adequacy, %	75 (58–91)

Data are median (1st–3rd quartile) or *n* (%). ACE: angiotensin-converting enzyme; ARB: angiotensin II receptor blocker; BMI: body mass index; BNP: B-type natriuretic peptide; CONUT: controlling nutritional status; eGFR: estimated glomerular filtration rate; GNRI: geriatric nutritional risk index; LVEF: left ventricular ejection fraction; MRA: mineralocorticoid receptor antagonist; NYHA: New York Heart Association.

**Table 2:** Characteristics of chronic HF patients with adequate calorie intake and those with inadequate calorie intake.

	Adequate Calorie Intake (*N* = 101)	Inadequate Calorie Intake (*N* = 44)	*p*-Value
Demographic findings:			
Age, yrs	68 (61–78)	65 (55–73)	0.04
Female	35 (35%)	9 (20%)	0.07
BMI, kg/m^2^	22.8 (20.3–26.1)	23.4 (20.7–25.4)	0.74
Upper arm circumference, cm	27.8 (24.7–30.0)	26.8 (24.9–29.4)	0.61
Thigh circumference, cm	44.4 (40.4–47.2)	43.9 (41.5–46.9)	0.99
NYHA functional class:			0.07
I–II	94 (93%)	36 (82%)	
III	7 (7%)	8 (18%)	
Primary cause of HF:			
Ischemic cause	33 (33%)	13 (30%)	0.71
Dilated cardiomyopathy	28 (28%)	17 (39%)	0.19
Others	40 (40%)	14 (32%)	0.37
Hypertension	55 (54%)	25 (57%)	0.79
Diabetes	21 (21%)	17 (39%)	0.02
Dyslipidemia	68 (67%)	32 (73%)	0.52
Echocardiographic findings:			
LVEF, %	49 (37–59)	34 (25–48)	<0.01
Laboratory measurements:			
Hemoglobin, g/dL	12.9 (11.7–14.2)	13.6 (12.4–14.4)	0.09
Serum albumin, g/dL	4.1(4.0–4.3)	4.3 (3.9–4.5)	0.39
eGFR, mL/min/1.73 m^2^	57.5 (42.9–71.1)	45.7 (34.5–56.5)	<0.01
HbA1c, %	5.8 (5.5–6.2)	5.9 (5.7–6.5)	0.17
Plasma BNP, pg/mL	153 (78–346)	156 (57–431)	0.88
Medications:			
ACE inhibitor or ARB	75 (74%)	35 (80%)	0.49
β-blocker	87 (86%)	41 (93%)	0.23
MRA	53 (52%)	30 (68%)	0.08
Statin	47 (47%)	22 (50%)	0.7
6-min walk test, m	435 (364–502)	424 (335–456)	0.15
Nutritional assessments:			
CONUT score	2 (1–2)	2 (1–3)	0.86
GNRI	106 (99–113)	107 (102–111)	0.54
Dietary calorie intake, kcal/day	1824 (1566–2276)	1145 (950–1308)	<0.01
Estimated calorie requirement, kcal/day	2238 (1913–2419)	2350 (2200–2469)	0.02
Dietary calorie intake adequacy, %	83 (73–99)	51 (42–57)	<0.01

Data are median (1st–3rd quartile) or n (%). Inadequate calorie intake was defined as <60% of estimated calorie requirement. ACE: angiotensin-converting enzyme; ARB: angiotensin II receptor blocker; BMI: body mass index; BNP: B-type natriuretic peptide; CONUT: controlling nutritional status; eGFR: estimated glomerular filtration rate; GNRI: geriatric nutritional risk index; LVEF: left ventricular ejection fraction; MRA: mineralocorticoid receptor antagonist; NYHA: New York Heart Association.

**Table 3:** Multivariate analysis of predictors of adverse clinical events including all-cause death and HF-related hospitalization in patients with chronic HF.

	Dietary Calorie IntakeAdequacy	OR	95% CI	*p*-Value
Inadequate calorie intake	<80%	2.16	0.33–14.2	0.42
	<70%	4.89	0.68–35.1	0.11
	<60%	7.39	1.02–53.5	0.04

As confounding factors that may influence patient’s dietary calorie intake, age, BMI, NYHA functional class III, diabetes, LVEF, serum albumin, eGFR, and log BNP were included in each analysis. OR: odds ratio; CI: confidence interval.

## Data Availability

Data supporting the findings of this work are available from the corresponding author upon reasonable request. All the data are obtained from subjects and are not publicly available due to ethical reasons.

## Supplementary Materials

The following are available online at https://www.mdpi.com/2072-6643/13/3/874/s1. Table S1: Daily intakes of foods and beverages estimated using a BDHQ, Table S2: Daily intakes of nutrients estimated using a BDHQ.
