# Supplementary material for: Impact of Inadequate Calorie Intake on Mortality and Hospitalization in Stable Patients with Chronic Heart Failure
Source: Nutrients. 2021 Mar 8;13(3):874. doi: 10.3390/nu13030874 (PMC7998469; doi:10.3390/nu13030874)
Supplement: Supplementary file 1 [file nutrients-13-00874-s001.pdf]

Supplementary Table S1. Daily intakes of foods and beverages estimated using a BDHQ

	Adequate calorie intake (n=101)	Inadequate calorie intake (n=44)	p–value
Low fat milk, g	0 (0–68)	0 (0–17)	0.18
Whole milk, g	59 (0–156)	11 (0–56)	0.04
Chicken, g	16 (7–36)	11 (5–27)	<0.01
Pork/beef, g	28 (13–36)	12 (6–28)	<0.01
Ham/sausage/bacon, g	5 (2–12)	2 (0–4)	<0.01
Squid/octopus/shrimp/shellfish, g	14 (6–30)	6 (6–14)	0.02
Small fish with bones, g	6 (0–22)	0 (0–5)	<0.01
Canned tuna, g	0 (0–4)	0 (0–3)	0.08
Dried fish/salted fish, g	26 (12–33)	11 (5–26)	<0.01
Oily fish, g	15 (7–34)	7 (6–17)	<0.01
Lean fish, g	26 (12–41)	12 (6–15)	<0.01
Egg, g	30 (21–60)	23 (9–27)	<0.01
Tofu, g	40 (18–79)	15 (13–36)	<0.01
Natto, g	0 (0–17)	0 (0–7)	0.18
Potatoes, g	46 (18–58)	18 (10–46)	<0.01
Pickled green leafy vegetables, g	0 (0–4)	0 (0–2)	0.25
Other pickled vegetables, g	2 (0–12)	1 (0–3)	0.08
Cabbage/Chinese cabbage, g	21 (15–48)	17 (6–40)	0.02
Green-leaved vegetables, g	30 (13–63)	8 (5–27)	<0.01
Carrots/pumpkin, g	19 (8–38)	15 (3–17)	<0.01
Japanese radish/turnip, g	25 (6–45)	10 (4–26)	0.01
Other root vegetables, g	34 (22–62)	26 (10–51)	0.03
Tomatoes, g	25 (6–51)	9 (4–23)	<0.01
Mushrooms, g	11 (4–18)	4 (2–12)	<0.01
Seaweeds, g	10 (4–14)	5 (2–11)	<0.01
Western-type confectioneries, g	5 (0–27)	5 (0–10)	<0.01
Japanese-type confectioneries, g	4 (0–8)	0 (0–4)	<0.01
Rice crackers, g	7 (3–21)	4 (0–4)	<0.01
Ice cream, g	9 (0–20)	8 (0–20)	0.42
Citrus fruits, g	37 (7–74)	10 (0–37)	<0.01
Persimmons/strawberries, g	7 (0–32)	0 (0–15)	0.03
Other fruits, g	37 (13–74)	14 (0–37)	0.01
Mayonnaise/dressing, g	5 (2–10)	2 (1–5)	<0.01
Bread, g	32 (12–65)	10 (4–46)	<0.01
Buckwheat noodles, g	12 (0–24)	10 (2–21)	0.37
Japanese wheat noodles, g	16 (7–46)	15 (8–21)	0.39

Chinese noodles, g	9 (0–17)	9 (0–18)	0.90
Spaghetti/macaroni, g	9 (0–18)	9 (0–11)	0.54
Green tea, g	150 (5–433)	124 (0–433)	0.57
Black tea/oolong tea, g	0 (0–54)	0 (0–12)	0.43
Coffee, g	124 (12–375)	23 (0–173)	0.02
Coke/soft drink, g	13 (0–82)	7 (0–33)	0.45
100% fruit and vegetable juice, g	0 (0–82)	0 (0–15)	0.06
Rice, g	270 (208–360)	208 (120–270)	<0.01
Miso soup, g	96 (54–139)	69 (55–121)	0.23

Data are median (1st–3rd quartile). Inadequate calorie intake was defined as <60% of estimated calorie requirement.

Supplementary Table S2. Daily intakes of nutrients estimated using a BDHQ

	Adequate calorie intake (n=101)	Inadequate calorie intake (n=44)	p–value
Total protein, g	74 (61–93)	43 (32–53)	<0.01
Animal protein, g	43 (33–60)	25 (16–34)	<0.01
Vegetable protein, g	29 (23–36)	17 (14–22)	<0.01
Total fat, g	51 (42–69)	28 (21–36)	<0.01
Animal fat, g	27 (18–33)	14 (9–20)	<0.01
Vegetable fat, g	25 (20–33)	15 (10–21)	<0.01
Carbohydrate, g	246 (210–289)	161 (110–192)	<0.01
Ash, g	19 (15–23)	11 (9–14)	<0.01
Sodium, g	4.1 (3.5–5.2)	2.6 (2.1–3.3)	<0.01
Potassium, g	2.7 (2.0–3.4)	1.5 (1.0–2.0)	<0.01
Calcium, mg	622 (446–767)	308 (229–415)	<0.01
Magnesium, mg	269 (212–326)	150 (110–193)	<0.01
Phosphorus, mg	1157 (906–1394)	639 (469–776)	<0.01
Iron, mg	7.9 (6.0–9.8)	4.5 (3.2–6.0)	<0.01
Zinc, mg	8.1 (6.7–9.8)	4.7 (3.8–6.1)	<0.01
Copper, mg	1.1 (0.9–1.4)	0.7 (0.6–0.9)	<0.01
Manganese, mg	3.3 (2.4–4.1)	2.3 (1.5–2.9)	<0.01
Vitamin A, μg	639 (491–944)	343 (224–517)	<0.01
Vitamin D, μg	16.8 (11.7–27.0)	8.9 (4.7–14.3)	<0.01
α–tocopherol, mg	7.5 (6.1–9.5)	4.6 (2.7–5.7)	<0.01
Vitamin K, μg	239 (149–379)	126 (78–218)	<0.01
Vitamin B1, mg	0.8 (0.6–1.0)	0.4 (0.3–0.6)	<0.01
Vitamin B2, mg	1.4 (1.1–1.7)	0.7 (0.6–1.0)	<0.01
Niacin, mg	18.0 (13.9–24.3)	10.4 (7.1–13.7)	<0.01
Vitamin B6, mg	1.3 (1.1–1.8)	0.8 (0.6–1.0)	<0.01
Vitamin B12, μg	11.5 (7.6–16.7)	6.0 (3.4–9.7)	<0.01
Folate, μg	339 (245–442)	212 (150–272)	<0.01
Pantothenic acid, mg	6.5 (5.6–7.9)	3.7 (2.8–4.6)	<0.01
Vitamin C, mg	126 (87–166)	85 (50–104)	<0.01
α–carotene, μg	358 (152–714)	285 (64–325)	<0.01
β–carotene, mg	3.2 (1.7–4.9)	1.2 (0.7–2.7)	<0.01
Cryptoxanthin, μg	297 (121–628)	125 (27–279)	<0.01
β–tocopherol, mg	0.3 (0.3–0.4)	0.2 (0.1–0.3)	<0.01
γ–tocopherol, mg	11.7 (9.1–14.9)	7.2 (4.3–10.2)	<0.01
σ–tocopherol, mg	2.8 (2.1–3.7)	1.7 (1.0–2.2)	<0.01
Saturated fat, g	14.0 (10.7–18.1)	6.8 (5.4–9.9)	<0.01

Monounsaturated fat, g	17.4 (14.9–24.0)	9.9 (7.2–13.4)	<0.01
Polyunsaturated fat, g	12.6 (9.7–15.1)	7.5 (4.6–9.9)	<0.01
Cholesterol, mg	412 (260–543)	202 (128–279)	<0.01
Total dietary fiber, g	12.2 (8.9–15.3)	6.9 (5.2–9.3)	<0.01
Soluble dietary fiber, g	3.0 (2.2–3.9)	1.7 (1.3–2.4)	<0.01
Insoluble dietary fiber, g	9.0 (6.5–11.0)	5.2 (3.7–6.8)	<0.01
Salt, g	10.2 (8.8–13.3)	6.6 (5.4–8.3)	<0.01
Sucrose, g	9.4 (5.0–15.1)	3.9 (1.7–10.1)	<0.01

Data are median (1st–3rd quartile). Inadequate calorie intake was defined as <60% of the estimated calorie requirement.
